# Associations between antihypertensive drug-related gene polymorphisms and cardiovascular outcomes in hypertensive patients: a gene-drug interaction study

**DOI:** 10.1038/s41598-026-48528-w

**Published:** 2026-04-12

**Authors:** Zetong Li, Huixia Liu, Mengshi Chen, Jing Deng, Ziyu Ma, Ge Huang, Cheng Li, Meng Su, Hua Zhong

**Affiliations:** 1 Xiangya School of Public Health, Central South University, Changsha, China; 2 Disease Prevention and Control Institute, China Railway Urumqi Group Co., Ltd, Urumqi, China; 3 Hunan Provincial Key Laboratory of Clinical Epidemiology, Central South University, Changsha, China; 4 Department of Cardiology, Xiangya Hospital, Central South University, Changsha, 410008 China

**Keywords:** Hypertension, Antihypertensive drugs, Genetic polymorphism, Cardiovascular and cerebrovascular events, Cardiology, Diseases, Drug discovery, Genetics, Medical research

## Abstract

Antihypertensive therapy is pivotal in preventing cardiovascular events, yet treatment efficacy varies significantly among individuals due to genetic polymorphisms.This study aimed to investigate the association between specific antihypertensive drug-related gene polymorphisms, the use of corresponding sensitive drugs, and the risks of stroke and coronary heart disease (CHD) in a community-based hypertensive population. A cross-sectional study was conducted among 29,662 hypertensive patients from primary care centers in Changsha County, China. Seven gene loci (ACE(I/D), CYP2C9*3,AGTR1(1166 A > C), CYP2D6*10,ADRB1(1165G > C), CYP3A5*3, and NPPA(2238T > C))were genotyped.Patients were stratified into sensitive or non-sensitive genotype groups. Logistic regression and interaction analysis were employed to assess gene-drug interactions on cardiovascular outcomes. The prevalence of stroke and CHD was 2.8% and 19.5%, respectively. Carriers of sensitive genotypes for AGTR1, CYP2D6*10, and ADRB1 taking sensitive drugs exhibited a significantly lower risk of stroke (ORs: 0.39, 0.67, 0.68; all *P* < 0.01). Similarly, sensitive genotype carriers for CYP2D6*10, CYP3A5*3, and NPPA taking sensitive drugs had a reduced risk of CHD (ORs: 0.92, 0.88, 0.53; all *P* < 0.05). A significant additive interaction was identified between AGTR1(1166 A > C) and sensitive drug use on CHD risk (AP = 0.08). Pharmacogenomics-guided antihypertensive therapy is associated with a reduced risk of stroke and CHD in hypertensive patients. The interaction between AGTR1(1166 A > C) genotype and drug use underscores the potential of personalized medicine in optimizing cardiovascular disease prevention strategies in primary care.

## Introduction

Cardiovascular disease and stroke are recognized as the first and second leading causes of death globally, respectively, posing a severe dual threat to public heal^1^. In 2019, cardiovascular and cerebrovascular diseases accounted for 32% of global deaths. Among these, 85% of fatalities resulted from coronary heart disease and stroke^2^. From 1990 to 2019, the absolute incidence of stroke increased by 70% globally, while its prevalence rose by 85%^3^. Currently, CHD and stroke are the leading causes of death among adults in China^4,5^.

Hypertension is a key risk factor for severe cardiovascular diseases such as stroke and coronary heart disease. Epidemiological studies indicate that blood pressure levels are positively correlated with the risk of stroke and coronary heart disease^6,7^. The 2024 WHO report indicates that approximately 1.3 billion people worldwide have hypertension, and this condition is responsible for about 54% of stroke deaths and 47% of coronary heart disease deaths^8^. China’s 2023 chronic disease surveillance data indicate that hypertensive patients face a 4.2- to 5.1-fold increased risk of stroke and a 2.9- to 3.4-fold higher risk of coronary heart disease compared to normotensive individuals^9^. Although antihypertensive drug therapy effectively reduces the risk of cardiovascular and cerebrovascular events, the treatment attainment rate among community-dwelling hypertensive patients in China remains below 25%^10–12^, contributing to persistently high mortality rates from these diseases.

The prevalence of hypertension among adults in China is 27.9%^13^. Clinically, approximately 30% to 50% of patients experience poor blood pressure control due to individual differences in drug response(such as insufficient efficacy or adverse reactions)^14^.The traditional ‘trial-and-error approach,’ based on guideline recommendations and clinical experience, may reduce patient compliance and potentially delay optimal disease management or contribute to disease progression. Pharmacogenomics studies suggest that individual variations in antihypertensive drug efficacy are closely linked to genetic variations^15^. For instance, reports indicate that among patients receiving ACEI therapy, those with the ACE (I/D) DD genotype show greater blood pressure reduction compared to those with ID/II genotypes (*p* < 0.05)^16^. Similarly, CYP2C9*3 carriers have been observed to achieve greater blood pressure reduction with ARB therapy^17^,and the AGTR1 (1166 A > C) C allele has been associated with enhanced systolic blood pressure lowering in response to ARBs^18,19^. Other studies have reported that polymorphisms in the β1-adrenergic receptor gene influence metoprolol efficacy^20^, while CYP3A5*3 and NPPA(2238T > C) gene polymorphisms respectively affect the antihypertensive efficacy of CCBs and diuretics^21,22^. Based on this, the “sensitive genotype-sensitive drug” model—selecting drugs with theoretically superior efficacy based on patient genotypes—has been proposed as a potential strategy for precision treatment of hypertension^23^. However, existing studies have limitations. First, most research focuses solely on blood pressure control rather than cardiovascular and cerebrovascular events as endpoints^24–26^. Second, given the limited number of clinical studies conducted in the Chinese population, existing evidence remains insufficient to form authoritative conclusions guiding clinical practice for Chinese hypertensive patients^27^. Furthermore, systematic evaluations of gene-drug interactions are lacking, with particularly limited evidence on additive interactions^7^. Gene-drug interaction research holds significant value. From a biological mechanism perspective, drug-metabolizing enzyme genes CYP2C9 and CYP2D6 influence drug concentrations, while receptor genes AGTR1 and ADRB1 determine drug sensitivity. These two gene types jointly regulate drug efficacy^15,19,20^. Clinical data suggest that response variations to the same drug among patients with different genotypes can reach 3–5 fold differences^28^. Precise identification of drug-sensitive genotypes could help avoid ineffective treatment and improve resource utilization^29^. Therefore, conducting gene-drug association studies with cardiovascular and cerebrovascular events among Chinese hypertensive populations holds significant implications for the prevention and treatment of cardiovascular and cerebrovascular diseases. Leveraging the Changsha County Hypertension Patient Management System in Hunan Province, we conducted follow-up registrations of hypertension patients’ conditions and genetic test results. This enabled us to investigate the relationship between antihypertensive drug use and stroke/CHD outcomes across different genotypes. Our study clarifies the impact of common drug-sensitive genotypes and their corresponding sensitive drugs on stroke and CHD endpoints in Chinese hypertensive patients. This provides valuable preliminary evidence supporting the potential of genotype-guided antihypertensive therapy in the Chinese population, warranting further investigation in prospective studies.

## Materials and methods

### Study population

The study initially screened 33,210 individuals registered in the Changsha County Hypertension Management System, from January 1, 2012, to January 1, 2018. After excluding 3,511 individuals who were not diagnosed with hypertension by physicians and 37 individuals lacking genetic information, a final cohort of 29,662 hypertensive patients was included.

Inclusion criteria: ①Participants met the office blood pressure measurement criteria in the Chinese Hypertension Prevention and Treatment Guidelines, with three blood pressure readings taken on non-consecutive days showing seated systolic blood pressure (SBP) ≥ 140 mmHg and/or diastolic blood pressure (DBP) ≥ 90 mmHg;② Previously diagnosed with hypertension;③Currently using antihypertensive medications;④Complete registration records and informed consent obtained.

Exclusion Criteria: ①Individuals with registration records but lacking blood pressure measurement records;②Individuals lacking genetic information.

### Data sources

All patient data were obtained from the hypertension management module of the aforementioned National Basic Public Health Service Project Management Information System, which records medical and follow-up information for all registered hypertensive patients. Each hypertensive patient underwent four annual in-person follow-ups and one physical examination. Diagnoses of stroke, coronary heart disease, and other cardiovascular events were confirmed by higher-level hospitals.

This study was reviewed by the Ethics Committee of the Xiangya School of Public Health, Central South University (Approval No.: XYGW-2022-73).All methods were performed in accordance with the Declaration of Helsinki.

### Data collection

#### General Information

Basic information, medical history, family history, and antihypertensive medication use (ACEI, ARB, BB, CCB, diuretics) were collected by family physicians and public health personnel at community health service centers using standardized questionnaires.

(1) Basic information: Gender, age, height, weight, marital status, educational attainment, etc.

(2) Medical History: Current presence of diabetes and chronic obstructive pulmonary disease (COPD);

(3) Family History: Includes family history of hypertension, diabetes, coronary heart disease, and stroke;

(4) Antihypertensive Medication Use: Community workers recorded the patient’s use of five major classes of antihypertensive drugs—angiotensin-converting enzyme inhibitors (ACEIs), angiotensin II receptor blockers (ARBs), beta-blockers (BBs), calcium channel blockers (CCBs), and diuretics—every three months.

#### Physical examination

Conducted by professionally trained personnel at the community health center, primarily including height, weight, and blood pressure measurements. Specific testing methods are as follows:

(1) Height: Measured using a domestically produced column-type height/sitting height gauge by professionally trained staff. The measuring instrument must be inspected and calibrated before use. Readings are accurate to 0.1 cm, with two repeated measurements taken and the average recorded.

(2) Weight: Measured using electronic scales by professionally trained staff. Scales undergo verification and calibration before use. Readings are accurate to 0.1 kg.

(3) Blood Pressure: After subjects are diagnosed with hypertension, community staff measure and record blood pressure every three months. Specific measurement method: Trained and certified staff use calibrated standard electronic automatic sphygmomanometers. Measurements are taken three times, with at least two minutes between each reading. The average of the three readings serves as the individual’s blood pressure value.

### Genetic Testing

#### Selection of gene polymorphisms and definition of gene classification

By reviewing relevant literature and utilizing Hunan Honghao Biomedical Co., Ltd.‘s “Genotyping Chip for Individualized Hypertension Drug Therapy“^26^, seven gene polymorphisms were detected using the PCR-fluorescent probe method. Specifically, these include^1^: Angiotensin-Converting Enzyme Inhibitor (ACEI) Target Receptor Gene: ACE (I/D) (rs4646994)^2^; Angiotensin Receptor Blocker (ARB) target receptor gene: AGTR1 (1166 A > C) (rs5186), Metabolizing Enzyme Gene CYP2C9*3 (*1, *3)^3^; Beta-adrenergic receptor blocker (β-blocker) target receptor gene: ADRB1 (1165G > C) (rs1801253), metabolizing enzyme gene: CYP2D6*10 (*1, *10)^4^; Calcium channel blocker (CCB) metabolizing enzyme gene CYP3A5*3 (*1, *3)^5^; Diuretic receptor target gene: NPPA (2238T > C) (rs5063). Reference: “Guidelines for Rational Use of Drugs in Hypertension (2nd Edition)“^23^, drug metabolism enzymes, and the “Guidelines for Drug Target Gene Detection Technology (Trial)“^30^. We have compiled the names, functional significance, and associated medications for seven genetic mutations across five major drug classes. Based on the differential effects of genetic polymorphisms on the action (metabolism) of corresponding antihypertensive drugs, and in combination with the actual genotype frequencies detected in this study, the following genes associated with antihypertensive drugs are classified into sensitive (S) and non-sensitive (N) types: ACE (I/D), CYP2C9*3, AGTR1 (1166 A > C), CYP2D6*10, ADRB1 (1165G > C), CYP3A5*3, and NPPA (2238T > C) were classified as sensitive (S) and non-sensitive (I). See Table 1 for details.

**Table 1:** Names, functional significance, gene classification and related drugs of hypertension drug metabolism-related gene mutations.

Drug	Representative Drugs	Gene	Target Site	Genotype	Functional Significance	Sensitive Genotype (S)
ACEI	Benazepril, Enalapril, Captopril	*ACE*(I/D)	rs4646994	II	Angiotensin-Converting Enzyme Activity Normal	No
				ID	Slightly elevated angiotensin-converting enzyme activity	Yes
				DD	Elevated angiotensin-converting enzyme activity	Yes
		CYP2C9*3	*1,*3	*1/*1	Normal metabolic function	No
				1/*3	Slightly reduced metabolism	Yes
				*3/*3	Moderately slow metabolic function	Yes
ARB	Irbesartan, Valsartan, Candesartan, Losartan	*AGTR1(*1166 A > C)	rs5186	AA	Angiotensin II receptor activity normal	No
				AC	Slightly elevated angiotensin II receptor activity	Yes
				CC	Elevated angiotensin II receptor activity	Yes
BB	Propranolol, Apraclonidine, Atenolol	CYP2D6*10	*1,*10	*1/*1	Normal Metabolic Function	No
				1/*10	Slightly reduced metabolism	Yes
				*10/10	Moderately slow metabolic function	Yes
		*ADRB1*(1165G > C)	rs1801253	GG	Normal receptor sensitivity	No
				GC	Slightly increased receptor sensitivity	No
				CC	Enhanced receptor sensitivity	Yes
CCB	Nifedipine, Amlodipine, Carvedilol	CYP3A5*3	*1,*3	*1/*1	Normal metabolic function	No
				*1/*3	Slightly reduced metabolism	Yes
				*3/*3	Moderately slow metabolic function	Yes
Diuretics	Hydrochlorothiazide, Amiloride	*NPPA* (2238T > C)	rs5063	TT	Normal receptor sensitivity	No
				TC	Slightly increased receptor sensitivity	Yes
				CC	Enhanced receptor sensitivity	Yes

Note: ACEI refers to angiotensin-converting enzyme inhibitor, ARB refers to angiotensin II receptor antagonist, BB refers to beta-blocker, CCB refers to calcium channel blocker.

#### Genetic polymorphism detection

From January 1, 2017, to January 1, 2018, oral mucosal exfoliated cells were collected from study subjects using buccal swabs, and DNA was extracted from these cells^31^. Genomic DNA was extracted using a commercial nucleic acid isolation kit (Promega, USA) and stored at −80℃. Subsequently, PCR-fluorescent probe technology was employed to detect polymorphisms in the ACE (I/D), CYP2C9*3, AGTR1 (1166 A > C), CYP2D6*10, ADRB1 (1165G > C), CYP3A5*3, and NPPA (2238T > C) gene polymorphisms (chips, sequencing primers, and other reagents for antihypertensive drug-related gene detection were provided by Hunan Honghao Biopharmaceutical Co., Ltd.). PCR conditions: 95 °C pre-denaturation for 2 min; denaturation at 95 °C for 40 min; 40 °C annealing at 60 °C; 40 °C extension at 72 °C; 40 cycles total; final extension at 72 °C for 5 min. Scanning performed using a GenePix 4100 A scanner with quantitative analysis conducted via its integrated GenePix 6.0 software. Subsequently, genotype interpretation was performed using analytical software based on preset Cuff values.

### Variable definitions

#### Definition of related indicators

Body Mass Index (BMI): BMI categories were defined according to the Working Group on Obesity in China criteria: underweight: <18.5 kg/m², normal: 18.5–24 kg/m², overweight: ≥24 kg/m².

#### Definition of outcome events

Blood Pressure Targets: Refer to the “Guidelines for the Prevention and Treatment of Hypertension 2018.” For general hypertensive patients, targets are < 140/90 mmHg; for hypertensive patients with diabetes, < 130/80 mmHg; for elderly patients aged 65 and above, < 150/90 mmHg.

Stroke: Refer to the diagnostic criteria for stroke in the “Guidelines for the Prevention and Treatment of Cerebrovascular Diseases (2024 Edition)“^32^. Specific diagnoses are conducted by secondary and tertiary hospitals.

Coronary Heart Disease: Diagnosis meets the criteria outlined in the Chinese Expert Consensus on Diagnosis and Treatment of Coronary Artery Microvascular Disease (2023 Edition)^33^. Specific diagnosis is conducted by secondary and tertiary hospitals.

### Statistical analysis

Statistical analyses were performed using SPSS 24.0 and R 4.3.0. Categorical variables were summarized as frequencies and percentages, and group comparisons were conducted using the χ² test.Two-sided P-values were used throughout, with *P* < 0.05 considered statistically significant. To account for multiple comparisons across the seven gene loci, Bonferroni correction was applied; a corrected P-value threshold of 0.007 (0.05/7) was used to determine statistical significance for the primary gene–drug interaction analyses.Univariate and multivariate logistic regression models were employed to estimate odds ratios (ORs) and 95% confidence intervals (CIs). Multivariate models were adjusted for potential confounders, including age, sex, body mass index (BMI), marital status, educational attainment, history of diabetes, history of chronic obstructive pulmonary disease (COPD), and family history of hypertension, coronary heart disease, and stroke. These covariates were selected based on their established associations with cardiovascular outcomes in the literature.Additive interactions between sensitive genotypes and corresponding drug use were evaluated using three indices: the relative excess risk due to interaction (RERI), the attributable proportion due to interaction (AP), and the synergy index (S). These measures were calculated using cross-product analysis with the “survival,” “epiR,” and “boot” packages in R 4.3.0.

## Results

### General characteristics of study participants

**Fig. 1:**
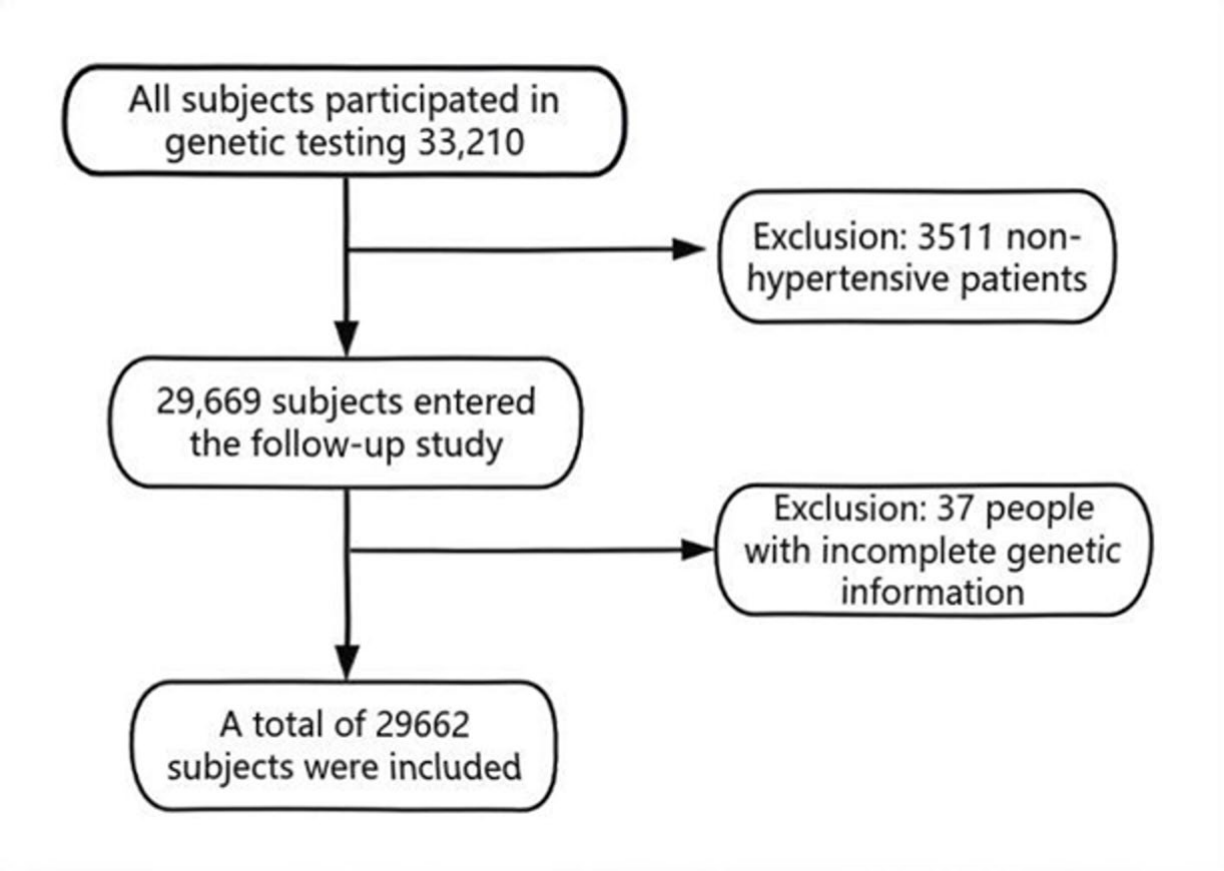
Study subject selection flowchart.

The system registered a total of 33,210 potential hypertensive patients (including those with undiagnosed elevated blood pressure). After excluding 3,511 non-hypertensive patients (diagnosed as non-hypertensive by physicians) and 37 individuals lacking genetic information, the final cohort comprised 29,662 hypertensive patients (59.9% female, 40.1% male), as shown in Fig. 1. Among this cohort, 50.0% were aged ≥ 65 years. Normal BMI and overweight individuals accounted for 50.8% and 45.3% of the total population, respectively. There were 816 stroke patients, representing a stroke prevalence of 2.8%, and 5,780 coronary heart disease (CHD) patients, with an overall CHD prevalence of 19.5%. Additional general characteristics are detailed in Table 2.

**Table 2:** Comparison of general characteristics among study participants (*N* = 29,662).

Item	Category	Number (*n*)	Percentage (%)
Gender	Male	11,903	40.1
	Female	17,759	59.9
Age (years)	< 65	14,656	49.5
	≥ 65	15,006	50.5
BMI (kg^/m²^)	< 18.5	1150	3.9
	18.5–24	15,066	50.8
	≥ 24	13,446	45.3
Household Registration Type	Rural	28,026	94.5
	Urban	1636	5.5
Marital Status	Single	600	2.1
	Married	25,935	87.4
	Divorced/Widowed	3,127	10.5
Educational Attainment	Elementary school or below	16,065	54.2
	Junior High School	11,507	38.8
	High school and above	2090	7.0
History of diabetes	No	24,319	82.0
	Yes	5343	18.0
History of chronic obstructive pulmonary disease	No	29,310	98.8
	Yes	352	1.2
Family history of hypertension	No	26,109	88.0
	Yes	3553	12.0
Family history of coronary heart disease	No	28,564	96.3
	Yes	1098	3.7
Family history of stroke	No	29,291	98.7
	Yes	371	1.3
Family history of diabetes	No	29,202	98.4
	Yes	460	1.6
Total		29,662	100.0

### Distribution of hypertension-related genes

The distribution of sensitive genotypes is detailed in Table 3.

**Table 3:** Distribution frequency of hypertension medication-related gene genotypes.

Gene	Sensitive Genotype	*n*(%)	Non-sensitive Genotype	*n*(%)
*ACE*(I/D)	ID + DD	15,703 (52.9)	II	13,959 (47.1)
CYP2C9*3	1/*3+*3/*3	2457 (8.3)	*1/*1	27,205 (91.7)
*AGTR1*(1166 A > C)	AC + CC	3203 (10.8)	AA	26,459 (89.2)
CYP2D6*10	1/*10+*10/*10	23,407 (78.9)	*1/*1	6255 (21.1)
*ADRB1*(1165G > C)	CC	16,472 (55.5)	GC + GG	13,190 (44.5)
CYP3A5*3	1/*3+*3/*3	2784 (9.4)	*1/*1	26,878 (90.6)
*NPPA* (2238T > C)	TC + CC	570 (1.9)	TT	29,092 (98.1)

### Study on the association between sensitive drug use and blood pressure control in patients with sensitive genotypes

This study systematically evaluated the relationship between seven key antihypertensive drug-response genesand the efficacy of their corresponding drug classes. Data indicate a significant statistical association between specific genotypes and blood pressure control rates when patients received corresponding pharmacogenetically guided drugs. Multiple gene-drug combinations, such as ACE(I/D) and CYP3A5*3 (associated with systolic blood pressure control), and NPPA (associated with diastolic blood pressure control), were associated with significantly higher blood pressure control rates. Details are presented in Table 4.

**Table 4:** Blood pressure control rates by genotype and antihypertensive drug use (*n* = 29,662).

Gene	Drug	Historical Systolic Blood Pressure Attainment		χ^2^	*P*	Historical Diastolic Blood Pressure Control		χ^2^	*P*
	Taking	No	Yes			No	Yes		
***ACE(I/D)***	ACEI								
II(-)	No	5475(56.5)	4214 (43.5)	17.11	< 0.001	3830 (39.5)	5859 (60.5)	16.80	< 0.001
	Yes	2572 (60.3)	1696 (39.7)			1845 (43.2)	2423(56.8)		
ID(+)	No	4865 (55.6)	3878(44.4)	25.18	< 0.001	3449 (39.4)	5294 (60.6)	13.37	< 0.001
	Yes	2359 (60.4)	1545 (39.6)			1675 (42.9)	2229 (57.1)		
DD(+)	No	1178(54.8)	973(45.2)	2.88	0.09	838(39.0)	1313(61.0)	0.368	0.54
	Yes	527(58.1)	380(41.9)			364(40.1)	543(59.9)		
Total	No	11,518(56.0)	9,065 (44.0)	44.63	< 0.001	8117 (39.4)	12,466 (60.6)	29.36	< 0.001
	Yes	5458 (60.1)	3621 (39.9)			3884 (42.8)	5195 (57.2)		
***CYP2C9***	ARB								
*1/*1(-)	No	14,221 (57.5)	10,507 (42.5)	8.52	0.004	9937 (40.2)	14,791 (59.8)	7.56	< 0.01
	Yes	1348 (54.5)	1127(45.5)			1065 (43.0)	1410 (57.0)		
*1/*3(+)	No	1260 (57.8)	919 (42.2)	2.28	0.131	886 (40.7)	1293 (59.3)	0.749	0.387
	Yes	109 (52.4)	99 (47.6)			91 (43.7)	117 (56.3)		
*3/*3(+)	No	36(54.5)	30(45.5)	0.624^a^	0.399	21(31.8)	45(68.2)	0.593^a^	0.399
	Yes	3(75.0)	1(25.0)			2(50.0)	2(50.0)		
Total	No	15,518 (57.5)	11,457 (42.5)	10.17	< 0.01	10,845 (40.2)	16,130(59.8)	8.49	< 0.01
	Yes	1460 (54.3)	1227 (45.7)			1158 (43.1)	1529 (56.9)		
***AGTR1***	ARB								
AA(-)	No	13,881 (57.6)	10,203(42.4)	7.716	< 0.01	9696 (40.3)	14,388 (59.7)	6.534	< 0.05
	Yes	1297 (54.7)	1075 (45.3)			1019 (43.0)	1353 (57.0)		
AC(+)	No	1531 (56.2)	1193 (43.8)	1.47	0.225	1073 (39.4)	1651 (60.6)	2.853	0.091
	Yes	156 (52.5)	141 (47.5)			132 (44.4)	165 (55.6)		
CC(+)	No	103(62.8)	61(37.2)	3.88	< 0.05	74(45.1)	90(54.9)	0.255	0.613
	Yes	7(38.9)	11(61.1)			7(38.9)	11(61.1)		
Total	No	15,518 (57.5)	11,457 (42.5)	10.17	< 0.01	10,845 (40.2)	16,130(59.8)	8.49	< 0.01
	Yes	1460 (54.3)	1227 (45.7)			1158 (43.1)	1529 (56.9)		
***CYP2D6***	BBs								
*1/*1(-)	No	3271 (57.1)	2459(42.9)	0.052	0.82	2235 (39.0)	3495 (61.0)	2.429	0.119
	Yes	297 (56.6)	228(43.4)			223 (42.5)	302(57.5)		
*1/*10(+)	No	6737 (57.4)	5005 (42.6)	2.385	0.122	4809 (41.0)	6933 (59.0)	0.891	0.345
	Yes	585 (54.9)	480 (45.1)			452 (42.4)	613 (57.6)		
*10/*10(+)	No	5578(57.9)	4054(42.1)	11.73	< 0.001	3890(40.4)	5742(59.6)	0.081	0.775
	Yes	479(52.1)	441(47.9)			376(40.9)	544(59.1)		
Total	No	15,616 (57.5)	11,532 (42.5)	10.52	< 0.01	10,952 (40.3)	16,196 (59.7)	2.05	0.152
	Yes	1362 (54.2)	1152 (45.8)			1051 (41.8)	1463 (58.2)		
***ADRB1***	BBs								
GC(-)	No	5882 (57.5)	4347 (42.5)	0.998	0.318	4113 (40.2)	6116 (59.8)	1.519	0.218
	Yes	553 (55.9)	437 (44.1)			418 (42.2)	572 (57.8)		
GG(-)	No	1021(56.3)	791(43.7)	0.303	0.582	693(38.2)	1119(61.8)	0.016	0.899
	Yes	86(54.1)	73(45.9)			60(37.7)	99(62.3)		
CC(+)	No	8713 (57.7)	6394 (42.3)	11.34	< 0.001	6146 (40.7)	8961 (59.3)	0.869	0.351
	Yes	723 (53.0)	642 (47.0)			573 (42.0)	792 (58.0)		
Total	No	15,616 (57.5)	11,532 (42.5)	10.52	< 0.01	10,952 (40.3)	16,196 (59.7)	2.05	0.152
	Yes	1362 (54.2)	1152 (45.8)			1051 (41.8)	1463 (58.2)		
***CYP3A5***	CCB								
*1/*1(-)	No	334(51.5)	314(48.5)	6.915	< 0.01	274 (42.3)	374 (57.7)	2.646	0.104
	Yes	1226 (57.4)	910 (42.6)			827 (38.7)	1309(61.3)		
*1/*3(+)	No	1463 (50.8)	1415 (49.2)	67.53	< 0.001	1078 (37.5)	1800 (62.5)	12.59	< 0.001
	Yes	5706(59.5)	3889 (40.5)			3949 (41.2)	5646 (58.8)		
*3/*3(+)	No	1690(51.7)	1578(48.3)	53.05	< 0.001	1290(39.5)	1978(60.5)	2.951	0.086
	Yes	6556(58.9)	4578(41.1)			4582(41.2)	6552(58.8)		
Total	No	3487 (51.3)	3,307 (48.7)	125.90	< 0.001	2642 (38.9)	4152 (61.1)	9.12	< 0.01
	Yes	13,491 (59.0)	9377 (41.0)			9361 (40.9)	13,507 (59.1)		
***NPPA***	Diuretics								
TT(-)	No	12,553(56.3)	9761(43.7)	34.22	< 0.001	8819(39.5)	13,495(60.5)	31.77	< 0.001
	Yes	4084 (60.3)	2692 (39.7)			2938 (43.4)	3838(56.6)		
CT(+)	No	201 (58.6)	142 (41.4)	0.411	0.521	129 (37.6)	214 (62.4)	0.012	0.921
	Yes	55 (55.0)	45 (45.0)			37 (37.0)	63 (63.0)		
CC(+)	No	71(71.0)	29(29.0)	6.62	< 0.05	68(68.0)	32(32.0)	8.60	< 0.01
	Yes	12(44.4)	15(55.6)			10(37.0)	17(63.0)		
Total	No	12,826 (56.4)	9932 (43.6)	21.33	< 0.001	9017 (39.6)	13,741 (60.4)	28.96	< 0.001
	Yes	4152 (60.1)	2757 (39.9)			2986 (43.3)	3918 (56.7)		

Note: a Fisher’s exact test was used due to small expected frequencies (< 5) in the corresponding subgroup. All other comparisons were performed using the χ² test.

### Study on the association between sensitive drug use and stroke/CHD in patients with sensitive genotypes

Among individuals with specific sensitive genotypes, the use of corresponding sensitive medications was associated with a significant reduction in the risk of stroke and coronary heart disease. Specifically, carriers of the AGTR1(1166 A > C), CYP2D6*10, and ADRB1(1165G > C) sensitive genotypes exhibited a significantly reduced risk of stroke when receiving these medications (*P* < 0.01). Furthermore, carriers of the CYP2D6*10, CYP3A5*3, and NPPA(2238T > C) sensitive genotypes demonstrated a reduced risk of coronary heart disease with sensitive drug therapy (*P* < 0.05). (Refer to Tables 5 and 6 for details).

**Table 5:** Association analysis of sensitive drug use and stroke in patients with sensitive genotypes.

Sensitive Genotype	Sensitive Medications Use	Univariate logistic regression analysis		Multivariate logistic regression analysis	
		OR (95% CI)	*P*	OR (95% CI)	*P*
***ACE*** **(I/D)**	ACEI				
ID + DD	No	1		1	
	Yes	0.86 (0.71–1.05)	0.14	0.85 (0.70–1.77)	0.10
CYP2C9*3	ARB				
*1/*3+*3/*3	No	1		1	
	Yes	0.72 (0.41–1.24)	0.23	0.72 (0.41–1.25)	0.24
***AGTR1*** **(1166 A > C)**	ARB				
AC + CC	No	1		1	
	Yes	0.40 (0.23–0.71)	0.02	0.39 (0.22–0.69)	< 0.01
CYP2D6*10	BB				
*1/*10+*10/*10	No	1		1	
	Yes	0.68 (0.57–0.81)	< 0.01	0.67 (0.56–0.80)	< 0.01
***ADRB1*** **(1165G > C)**	BB				
CC	No	1		1	
	Yes	0.69 (0.56–0.84)	< 0.01	0.68 (0.56–0.84)	< 0.01
CYP3A5*3	CCB				
*1/*3+*3/*3	No	1		1	
	Yes	1.10 (0.90–1.33)	0.36	1.07 (0.88–1.30)	0.52
***NPPA*** **(2238T > C)**	Diuretic				
CC + CT	No	1		1	
	Yes	0.51 (0.16–1.61)	0.25	0.58 (0.18–1.85)	0.36

Note: *Adjusted for age, sex, BMI, marital status, educational attainment, history of diabetes, history of COPD, and family history of hypertension, coronary heart disease, and stroke. ACEI denotes angiotensin-converting enzyme inhibitor, ARB denotes angiotensin II receptor antagonist, BB denotes beta-blocker, CCB denotes calcium channel blocker.

**Table 6:** Association Analysis of Sensitive Drug Use and Coronary Heart Disease in Patients with Sensitive Genotypes.

Sensitive Genotype	Sensitive Drug Use	Univariate logistic regression analysis		Multivariate logistic regression analysis	
		OR (95% CI)	*P*	OR^*^ (95% CI)	*P*
***ACE*** **(I/D)**	ACEI				
ID + DD	No	1		1	
	Yes	0.94 (0.87–1.01)	0.11	0.94 (0.86–1.02)	0.11
CYP2C9*3	ARB				
*1/*3+*3/*3	No	1		1	
	Yes	1.03 (0.84–1.26)	0.80	1.05 (0.85–1.30)	0.66
***AGTR1*** **(1166 A > C)**	ARB				
AC + CC	No	1		1	
	Yes	1.01 (0.84–1.21)	0.95	1.01 (0.83–1.22)	0.96
CYP2D6*10	BB				
*1/*10+*10/*10	No	1		1	
	Yes	0.93 (0.87–1.00)	0.05	0.92 (0.86–0.99)	0.02
***ADRB1*** **(1165G > C)**	BB				
CC	No	1		1	
	Yes	1.00 (0.92–1.09)	0.98	1.00 (0.92–1.09)	0.99
CYP3A5*3	CCB				
*1/*3+*3/*3	No	1		1	
	Yes	0.94 (0.87–1.02)	0.12	0.88 (0.81–0.95)	< 0.01
***NPPA*** **(2238T > C)**	Diuretics				
CC + CT	No	1		1	
	Yes	0.51 (0.32–0.81)	< 0.01	0.53 (0.32–0.88)	0.01

Note: *Adjusted for age, sex, BMI, marital status, educational attainment, history of diabetes, history of COPD, and family history of hypertension, coronary heart disease, and stroke. ACEI denotes angiotensin-converting enzyme inhibitor, ARB denotes angiotensin II receptor antagonist, BB denotes beta-blocker, CCB denotes calcium channel blocker.

### Gene-drug interaction analysis

No significant gene-drug interactions were observed for stroke as an outcome. For coronary heart disease (CHD) as an outcome, the AGTR1 (1166 A > C) gene showed an additive interaction with sensitive drug use on CHD risk (Tables 7 and 8).

**Table 7:** Gene-drug interaction in stroke onset.

Sensitive Genotype	Sensitive Drug Use	Control Group	Stroke Group	OR (95% CI)*	P*	RERI (95% CI)	AP (95% CI)	S (95% CI)
***ACE*** **(I/D)**	ACEI							
II(-)	No (-)	7791 (96.9)	539 (3.1)	1				
II(-)	Yes (+)	9380 (97.8)	213 (2.2)	1.03 (0.84–1.26)	0.79			
ID + DD(+)	No (-)	1547 (97.1)	46 (2.9)	1.01 (0.84–1.21)	0.91	0.02 (0.01–0.04)	0.02 (0.01–0.03)	1.66 (0.06–49.37)
ID + DD(+)	Yes(+)	846 (97.9)	18 (2.1)	0.83 (0.63–1.11)	0.21			
CYP2C9*3	ARB							
*1/*1(-)	No (-)	17,073 (96.9)	539 (3.1)	1				
*1/*1(-)	Yes (+)	9380 (97.8)	213 (2.2)	0.72 (0.62–0.85)	< 0.01			
*1/*3+*3/*3(+)	No (-)	1547 (97.1)	46 (2.9)	0.95 (0.70–1.29)	0.71	0.03 (−0.05 to 0.11)	0.04 (−0.08 to 0.16)	0.91 (0.71–1.07)
*1/*3+*3/*3(+)	Yes (+)	846 (97.9)	57 (2.5)	0.9 (0.56–1.75)	0.97			
***AGTR1*** **(1166 A > C)**	ARB							
AA(-)	No (-)	16,643 (97.0)	521 (3.0)	1				
AA(-)	Yes(+)	9079 (97.7)	216 (2.3)	0.77 (0.65–0.90)	< 0.01			
AC + CC (+)	No (-)	1977 (96.9)	64 (3.1)	1.04 (0.80–1.35)	0.79	0.01 (−0.05 to 0.07)	0.01 (−0.06-0.09)	0.95 (0.75–1.21)
AC + CC(+)	Yes (+)	1147 (98.7)	15 (1.3)	0.53 (0.29–0.95)	0.03			
CYP2D6*10	BB							
*1/*1(-)	No (-)	3982 (96.9)	126 (3.1)	1				
*1/*1(-)	Yes (+)	2103 (98.0)	44 (2.0)	0.66 (0.46–0.93)	0.02			
*1/*10+*10/*10(+)	No (-)	14,927 (96.9)	477 (3.1)	1.01 (0.83–1.24)	0.90	0.01 (−0.05 to 0.07)	0.02 (−0.08 to 0.12)	0.97 (0.81–1.41)
*1/*10+*10/*10(+)	Yes (+)	7834 (97.9)	169 (2.1)	1.03 (0.70–1.52)	0.89			
***ADRB1*** **(1165G > C)**	BB							
GC + GG(-)	No (-)	11,791 (97.4)	309 (2.6)	1				
GC + GG(-)	Yes (+)	1071 (98.3)	19 (1.7)	0.69 (0.56–0.85)	< 0.01			
CC(+)	No (-)	10,464 (96.7)	358 (3.3)	0.85 (0.72–1.01)	0.60	0.06 (0.01–0.11)	0.10 (−0.01 to 0.21)	0.87 (0.82–0.92)
CC(+)	Yes(+)	5520 (97.7)	130 (2.3)	0.93 (0.67–1.29)	0.67			
CYP3A5*3	CCB							
*1/*1(-)	No (-)	499 (97.5)	13 (2.5)	1				
*1/*1(-)	Yes (+)	2202 (96.9)	70 (3.1)	1.19 (0.65–2.17)	0.58			
*1/*3+*3/*3(+)	No (-)	4880 (97.5)	127 (2.5)	1.00 (0.56–1.79)	0.99	0.03 (−0.11 to 0.17)	0.02 (−0.07 to 0.11)	1.15 (0.88–1.50)
*1/*3+*3/*3(+)	Yes (+)	21,265 (97.2)	606 (2.8)	0.90 (0.48–1.70)	0.75			
***NPPA*** **(2238T > C)**	Diuretics							
TT(-)	No (-)	16,376 (96.8)	535 (3.2)	1				
TT(-)	Yes(+)	11,916 (97.8)	265 (2.2)	0.68 (0.59–0.97)	< 0.01			
CT + CC (+)	No (-)	336 (96.6)	12 (3.4)	1.10 (0.62–1.98)	0.74	−0.02 (−0.21 to −0.18)	−0.02 (−0.30 to 0.22)	1.07 (0.38–3.05)
CT + CC(+)	Yes (+)	218 (98.2)	4 (1.8)	0.80 (0.25–2.53)	0.70			

Note: *Adjusted for age, sex, BMI, marital status, educational attainment, history of diabetes, history of COPD, and family history of hypertension, coronary heart disease, and stroke. ACEI denotes angiotensin-converting enzyme inhibitor, ARB denotes angiotensin II receptor antagonist, BB denotes beta-blocker, CCB denotes calcium channel blocker.

**Table 8:** Gene-Drug Interactions in Coronary Heart Disease.

Sensitive Genotype	Sensitive Drug Use	Control Group	CHD Group	OR (95% CI)^*^	*P* ^*^	RERI (95% CI)	AP (95% CI)	S (95% CI)
***ACE*** **(I/D)**	ACEI							
II(-)	No (-)	6475 (80.8)	1540 (19.2)	1				
II(-)	Yes (+)	4825 (81.2)	1119 (18.8)	0.99 (0.91–1.08)	0.85			
ID + DD(+)	No (-)	7165 (79.7)	1827 (20.3)	1.07 (0.99–1.15)	0.10	0.08 (0.06–0.09)	0.07 (0.06–0.07)	2.27 (0.65–7.98)
ID + DD(+)	Yes(+)	5417 (80.7)	1294 (19.3)	0.95 (0.84–1.07)	0.40			
CYP2C9*3	ARB							
*1/*1(-)	No (-)	14,054 (79.8)	3558 (20.2)	1				
*1/*1(-)	Yes (+)	7859 (81.9)	1734 (18.1)	0.88 (0.82–0.94)	< 0.05			
*1/*3+*3/*3(+)	No (-)	1279 (80.3)	314 (19.7)	0.95 (0.83–1.09)	0.48	0.07 (0.06–0.07)	0.07 (0.05–0.09)	0.61 (0.36–1.01)
*1/*3+*3/*3(+)	Yes (+)	690 (79.9)	174 (20.1)	1.19 (0.95–1.49)	0.13			
***AGTR1*** **(1166 A > C)**	ARB							
AA(-)	No (-)	13,666 (79.6)	3499 (20.4)	1				
AA(-)	Yes (+)	7601 (81.8)	1694 (18.2)	0.88 (0.82–0.94)	< 0.01			
AC + CC (+)	No (-)	1667 (81.7)	374 (18.3)	0.89 (0.79–1.00)	0.06	**0.07 (0.06–0.08)**	**0.08 (0.06–0.10)**	**0.70 (0.57–0.86)**
AC + CC(+)	Yes(+)	948 (81.6)	214 (18.4)	1.13 (0.92–1.38)	0.26			
CYP2D6*10	BB							
*1/*1(-)	No (-)	3286 (80.0)	822 (20.0)	1				
*1/*1(-)	Yes (+)	1728 (80.5)	419 (19.5)	1.01 (0.93–1.09)	0.80			
*1/*10+*10/*10(+)	No (-)	12,361 (80.2)	3043 (19.8)	1.03 (0.96–1.11)	0.43	0.08 (0.06–0.09)	0.07 (0.06–0.07)	3.07 (0.29–32.54)
*1/*10+*10/*10(+)	Yes (+)	6507 (81.3)	1496 (18.7)	0.84 (0.74–0.95)	< 0.01			
***ADRB1*** **(1165G > C)**	BB							
GC + GG(-)	No (-)	6949 (80.0)	1741 (20.0)	1				
GC + GG(-)	Yes (+)	3695 (82.1)	805 (17.9)	0.97 (0.84–1.11)	0.63			
CC(+)	No (-)	8698 (80.4)	2124 (19.6)	1.01 (0.92–1.10)	0.86	0.07 (0.06–0.08)	0.07 (0.06–0.09)	−1.78 (Na)
CC(+)	Yes(+)	4540 (80.4)	1110 (19.6)	0.95 (0.82–1.11)	0.55			
CYP3A5*3	CCB							
*1/*1(-)	No(-)	408 (79.7)	104 (20.3)	1				
*1/*1(-)	Yes (+)	1807 (79.5)	465 (20.5)	0.95 (0.74–1.21)	0.67			
*1/*3+*3/*3(+)	No (-)	3997 (79.8)	1010 (20.2)	0.99 (0.78–1.25)	0.91	0.07 (0.05–0.08)	0.07 (0.05–0.08)	−0.04 (Na)
*1/*3+*3/*3(+)	Yes (+)	19,477 (80.7)	4666 (19.3)	0.92 (0.71–1.20)	0.54			
***NPPA*** **(2238T > C)**	Diuretics							
TT(-)	No (-)	13,459 (79.6)	3452 (20.4)	1				
TT(-)	Yes (+)	9958 (81.8)	2223 (18.2)	0.86 (0.81–0.92)	< 0.01			
CT + CC (+)	No (-)	271 (77.9)	77 (22.1)	1.12 (0.86–1.46)	0.40	0.05 (0.03–0.08)	0.05 (0.02–0.09)	−2.35 (Na)
CT + CC(+)	Yes (+)	194 (87.4)	28 (12.6)	0.62 (0.38–1.01)	0.06			

Note: *Adjusted for age, sex, BMI, marital status, educational attainment, history of diabetes, history of COPD, and family history of hypertension, coronary heart disease, and stroke. ACEI denotes angiotensin-converting enzyme inhibitor, ARB denotes angiotensin II receptor antagonist, BB denotes beta-blocker, CCB denotes calcium channel blocker.

## Discussion

As one of the most common and serious health problems worldwide, hypertension leads to high morbidity and mortality rates, making it a major public health issue affecting global health. Stroke and coronary heart disease, as the two most common complications of hypertension, pose a significant threat to human health. Therefore, improving the therapeutic efficacy of antihypertensive medications is of great public health significance. This study indicates that among hypertensive patients in Changsha County, Hunan Province, the prevalence rates of stroke and coronary heart disease were 2.75% and 19.5%, respectively. These findings are generally consistent with the stroke prevalence rate among hypertensive patients in Jiangsu Province^34^ and the results of a study by Xu et al.^35^ in a community in Yanbian. However, the stroke incidence in this region is lower than the 6.6% recorded in Jiading District, Shanghai^36^. This discrepancy may stem from differences in medical resources, as Shanghai’s extensive medical infrastructure enables more accurate diagnosis, which in turn may lead to the identification of more mild or asymptomatic cases.A cohort study from Ramathibodi Hospital in Thailand, involving a 10-year follow-up, reported similar rates of coronary heart disease incidence; however, the incidence of stroke was 7.4%, slightly higher than that in our cohort^37^. This may be due to a higher burden of comorbidities at baseline in the Thai cohort, as well as the fact that the follow-up period in our cohort was shorter than theirs.Current hypertension management remains primarily pharmacological, involving five major classes of medications: angiotensin-converting enzyme inhibitors (ACEIs), angiotensin II receptor blockers (ARBs), beta-blockers (BBs), calcium channel blockers (CCBs), and diuretics. However, there are significant individual variations in response to antihypertensive medications. Although drug therapy is the cornerstone of hypertension management, lifestyle modifications—including dietary changes, regular exercise, and weight control—are equally crucial for optimizing blood pressure control and reducing cardiovascular risk. Studies have shown that variations in drug efficacy are associated with genetic variations among individuals. This study reveals that, among patients carrying different sensitive gene variants, whether they take sensitive medications affects their risk of stroke and coronary heart disease.

This study revealed a significant association between specific genetic polymorphisms associated with antihypertensive drugs and blood pressure control. The results indicate that among patients taking ACEIs (angiotensin-converting enzyme inhibitors), there is a significant difference in systolic and diastolic blood pressure control rates between those with the non-sensitive ACE(I/D) genotype II(-) and those with the sensitive genotype ID + DD(+) (*P* < 0.01), with patients of the ID + DD genotype exhibiting a slightly higher rate of diastolic blood pressure control. Regarding the NPPA (2238T > C) gene variant, patients with the sensitive CT + CC(+) genotype receiving diuretic therapy had a higher diastolic blood pressure control rate than those with the non-sensitive TT(-) genotype. However, among patients with the CYP3A5 *3 genotype taking calcium channel blockers (CCBs), those with the sensitive genotype had lower systolic blood pressure control rates. Although the control rates for the mutant genotype appear lower numerically, the CYP3A5 *3 mutation slows drug metabolism, thereby increasing plasma drug concentrations and enhancing the antihypertensive effect of CCBs^38^.Combined with the significant differences revealed by the chi-square test, this suggests that individuals with slow metabolism may be more likely to reach the blood pressure-lowering threshold due to drug accumulation. This is consistent with the biological mechanism by which CYP3A5 enzyme activity regulates drug exposure levels^39^. These findings are consistent with the results of numerous domestic and international studies and provide new evidence for specific gene-drug interactions. Subsequently, we investigated the association between stroke and coronary heart disease and the use of sensitive medications among carriers of sensitive genotypes for various genes associated with antihypertensive drugs. After adjusting for confounding factors such as age, sex, BMI, marital status, and educational level, the results showed that participants carrying the AGTR1 (1166 A > C), CYP2D6*10, and ADRB1 (1165G > C) susceptibility genotypes and taking corresponding susceptible medications had a 32%–61% reduced risk of stroke (OR 0.39–0.68); those carrying the CYP2D6*10, CYP3A53, and NPPA(2238T > C) genotypes had an 8%–47% reduced risk of coronary heart disease (OR 0.53–0.92). This is consistent with findings from other studies: the AGTR1 (1166 A > C) AC + CC genotype group exhibited enhanced blood pressure-lowering effects when taking irbesartan (ARB)^27^, while the CYP2D6*10 allele may reduce enzyme activity, significantly delaying drug metabolism^40^. Elevated plasma concentrations may enhance the inhibitory effect on sympathetic nerve activity, thereby indirectly reducing the risk of coronary heart disease^41^. In addition, the CC allele of the ADRB1 (1165G > C) gene exhibits greater antihypertensive efficacy when used in combination with metoprolol (BB)^18^. These findings suggest that individual genotypes may play a role in modulating drug responses. When using sensitive drugs, susceptible genotypes help lower blood pressure by influencing drug metabolism and targeting, thereby reducing the risk of stroke and coronary heart disease. These findings provide preliminary evidence for the potential of “personalized medicine” strategies and warrant further validation^23^.

Our findings indicate that, when coronary heart disease is used as an outcome measure, carriers of the AGTR1 (1166 A > C) allele exhibit an additive interaction with ARBs in terms of reducing the risk of coronary heart disease (RERI = 0.07, 95% CI: 0.06–0.08; AP = 0.08, 95% CI: 0.06–0.10; S = 0.70, 95% CI: 0.57–0.86). The AGTR1 1166 C allele enhances the activity of the angiotensin II receptor, whereas ARBs exert their antihypertensive effects by blocking this receptor. When combined, the efficiency of receptor-drug binding increases, thereby further reducing the risk of CHD (AP = 0.08)^18^.No additive interactions between the ACE (I/D), CYP2C9*3, CYP2D6*10, ADRB1 (1165G > C), CYP3A5*3, and NPPA (2238T > C) genes and drug sensitivity were found regarding CHD risk. The CYP2D6*10, CYP3A5*3, and NPPA rs1805127 genetic loci were associated with a reduced risk of CHD. However, the interactions between these variants and their corresponding sensitive drugs did not reach statistical significance. The primary reason may be the small sample size in the low-frequency genotype subgroups: the CYP3A5*3 mutation frequency was 9.4% (*n* = 2,784 in the corresponding subgroup); the frequency of NPPA rs1805127 was only 1.9% (*n* = 570), far below the minimum sample size required to detect weak interactions (AP < 0.1). When testing interactions involving low-frequency variants, insufficient statistical power may lead to false-negative results^42^. Second, although CYP3A5 *3 affects the metabolism of calcium channel blockers (CCBs), compensatory activity of CYP3A4/5 may attenuate their synergistic effects^43^. In contrast, NPPA rs1805127 lowers blood pressure by promoting natriuretic peptide secretion rather than through drug interactions^22^. Similarly, although CYP2D6 *10 may slow the metabolism of ARBs, ARBs themselves reduce myocardial oxygen consumption by blocking AT1R, and thus may independently reduce the risk of coronary heart disease through different mechanisms^41,44^. A clinical trial involving 9,467 participants^25^ and a study by Anh N Do et al.^26^ both support our findings: neither the ACE (I/D) gene nor lipid-lowering drugs were associated with the risk of cardiovascular events, and no statistically significant gene-treatment interactions were observed. However, our findings differ from those of a study on the NPPA (2238T > C) variant. Lynch et al.^22^observed that, when using the diuretic chlorthalidone, the TT genotype of the NPPA (2238T > C) gene was associated with a higher risk of coronary heart disease compared to the CC + CT genotypes. Possible reasons for these discrepancies include^1^: Differences in clinical endpoints: This study focused primarily on CHD events, whereas Lynch’s study included composite endpoints such as hospitalization for heart failure^2^. Differences in study design: This study was a cross-sectional study, whereas the previous study was a prospective cohort study. Further research is needed to validate these observed differences.

The response to antihypertensive therapy is increasingly recognized as a polygenic trait, involving the cumulative effects of multiple genetic variants across drug-metabolizing enzymes, transporters, and target receptors. Despite the inherent complexity of antihypertensive drug response, candidate gene studies remain a valuable initial approach for investigating genotype–drug interactions. The selection of the seven gene loci in this study was grounded in robust biological plausibility and prior evidence of their roles in drug metabolism or target receptor function. This hypothesis-driven design enhances interpretability and reduces the risk of false-positive findings that may arise from agnostic approaches, particularly in the context of a large community-based cohort.

This study has several limitations. First, both stroke and CHD involve complex pathophysiological mechanisms. Hemorrhagic and ischemic strokes, as well as acute and chronic coronary syndromes, differ in pathogenesis, clinical presentation, and prognosis. The data used in this study could not distinguish these subtypes, precluding assessment of potential differences. Second, the study population consisted of hypertensive patients in Changsha County, Hunan Province, predominantly of Han ethnicity. Future validation across different regions, ethnicities, and races is warranted. What’s more, this study did not specifically account for apparent resistant hypertension, a condition defined as uncontrolled blood pressure despite the use of three or more antihypertensive agents. The potential impact of resistant hypertension on the observed associations between gene-drug interactions and outcomes cannot be excluded and warrants further investigation in future studies.Fourth, this study employed a candidate gene approach focusing on seven well-established polymorphisms based on prior knowledge. While this targeted strategy offers interpretability and statistical efficiency, the pharmacogenomics of antihypertensive drug response is increasingly recognized as polygenic, involving the cumulative effects of numerous genetic variants, each typically contributing a modest effect. Consequently, our candidate gene design may not fully capture the complex genetic architecture underlying treatment response and cardiovascular outcomes. Future studies incorporating genome-wide association studies (GWAS) or polygenic risk scores (PRS) are warranted to provide a more comprehensive understanding of the genetic determinants of antihypertensive drug efficacy.Additionally, hypertension and cardiovascular/cerebrovascular diseases may share some common risk factors. As a cross-sectional study, the temporal relationship between hypertension and these diseases remains uncertain, potentially introducing reverse causality. Nevertheless, our study included a large cohort, established the temporal sequence between genes and outcomes, and systematically examined the relationship between sensitive drug use and stroke/CHD in carriers of sensitive genotypes after adjusting for non-genetic factors associated with stroke and CHD.

## Conclusions

These findings suggest that the use of sensitive drugs according to genotype may be associated with improved blood pressure control and a lower risk of stroke and coronary heart disease among hypertensive patients. Specifically, among carriers of sensitive genotypes for AGTR1 (1166 A > C), CYP2D6*10, and ADRB1 (1165G > C), the risk of stroke was observed to be lower among those taking the corresponding sensitive drugs. Similarly, among carriers of sensitive genotypes for CYP2D6*10, CYP3A5*3, and NPPA (2238T > C), a lower risk of coronary heart disease was associated with sensitive drug use. An additive interaction between AGTR1 (1166 A > C) and ARB use was also observed for coronary heart disease risk. Further prospective studies are warranted to validate these associations.

## Data Availability

The data used to support the fndings of this study are available from the corresponding author upon request.

## Abbreviations

ACE: Angiotensin converting enzyme
ACEI: Angiotensin-Converting Enzyme Inhibitor
ADRB1: β1 adrenergic receptor
ANP: Atrial natriuretic peptide precursor
AP: Attributable Proportion due to Interaction
ARB: Angiotensin II receptor antagonist
ATR1: angiotensin II type 1 receptor
BB: Beta blocker
BMI: body mass index
CCB: calcium channel blocker
CHD: Coronary heart disease
CI: Confidence interval
CVD: cardiovascular disease
CYP2C9: cytochrome P4502C9
CYP2D6: cytochrome P4502D6
CYP3A5: cytochrome P450 3A5
CYP450: cytochrome P450
DBP: diastolic blood pressure
HWE: Hardy-Weinberg equilibrium
NPPA: Atrial natriuretic precursor A
M: mutant
MM: homozygous mutant
OR: Odds Ratio
RAS: Renin-angiotensin system
RERI: Relative Excess Risk of Interaction
S: Synergy Index
SBP: systolic blood pressure
W: wild type
WM: heterozygous mutant
WW: homozygous wildtype
